# Impact of urinary incontinence on the quality of life of individuals undergoing radical prostatectomy^*^

**DOI:** 10.1590/1518-8345.2757.3131

**Published:** 2019-03-18

**Authors:** Mariana Ferreira Vaz Gontijo Bernardes, Sabrina de Cássia Chagas, Lívia Cristina de Resende Izidoro, Denny Fabricio Magalhaes Veloso, Tânia Couto Machado Chianca, Luciana Regina Ferreira Pereira da Mata

**Affiliations:** 1 Universidade Federal de Minas Gerais, Escola de Enfermagem, Belo Horizonte, MG, Brazil.; 2 Universidade Federal de São João Del Rei, Campus Centro-Oeste Dona Lindu, Divinópolis, MG, Brazil.; 3 Universidade Federal de Goiás, Faculdade de Enfermagem, Goiânia, GO, Brazil.

**Keywords:** Quality of Life, Health Related Quality of Life, Urinary Incontinence, Prostatectomy, Nursing, Prostatic Neoplasms, Qualidade de Vida, Qualidade de Vida Relacionada à Saúde, Incontinência Urinária, Prostatectomia, Enfermagem, Neoplasias da Próstata, Calidad de Vida, Calidad de Vida Relacionada Con la Salud, Incontinencia Urinaria, Prostatectomía, Enfermería, Neoplasias de la Próstata

## Abstract

**Objective:**

to assess the level of urinary incontinence and its impact on the quality of life of patients undergoing radical prostatectomy.

**Method:**

cross-sectional study carried out with prostatectomized patients. The data were collected from the following instruments: sociodemographic questionnaire, Pad Test, International Consultation on Incontinence Questionnaire - Short Form and King Health Questionnaire. Data were submitted to descriptive and bivariate statistical analysis. The level of significance was set at 0.05.

**Results:**

a total of 152 patients participated, with a mean age of 67 years. Among incontinent patients, there was a predominance of mild urinary incontinence. Urinary incontinence had a very severe impact on the general assessment of quality of life in the first months and severe impact after six months of surgery. The greater the urinary loss, the greater the impact on the quality of life domains Physical Limitations, Social Limitations, Impact of Urinary Incontinence and Severity Measures. Most participants reported no erection after surgery and therefore did not respond to the question of the presence of urinary incontinence during sexual intercourse.

**Conclusion:**

the present study evidenced the occurrence of urinary incontinence after radical prostatectomy at different levels and its significant impact on the quality of life of men, which reveals the need of interventions for controlling it.

## RESUMO

**Objetivo:**

avaliar o nível de incontinência urinária e o seu impacto na qualidade de vida de pacientes submetidos à prostatectomia radical.

**Método:**

estudo transversal, realizado com pacientes prostatectomizados. Os dados foram coletados a partir dos seguintes instrumentos: questionário sociodemográfico, *Pad Test*, *International Consultation on Incontinence Questionnaire - Short Form e King Health Questionnaire*. Os dados foram submetidos à análise estatística descritiva e bivariada. O nível de significância adotado foi de 0,05.

**Resultados:**

participaram 152 pacientes, com idade média de 67 anos. Entre os pacientes incontinentes, houve predomínio da incontinência urinária leve. A incontinência urinária causou impacto muito grave na avaliação geral da qualidade de vida nos primeiros meses e grave após seis meses de cirurgia. Quanto maior a perda urinária, maior impacto nos domínios da qualidade de vida Limitações Físicas, Limitações Sociais, Impacto da Incontinência Urinária e Medidas de Gravidade. A maioria dos participantes relatou ausência de ereção após a cirurgia e por isso não respondeu à questão referente à presença de incontinência urinária durante a relação sexual.

**Conclusão:**

o estudo evidenciou a ocorrência da incontinência urinária após prostatectomia radical em diferentes níveis e o seu impacto significativo na qualidade de vida dos homens merecendo intervenções para o seu controle.

## RESUMEN

**Objetivo:**

evaluar el nivel de incontinencia urinaria y su impacto en la calidad de vida de pacientes sometidos a la prostatectomia radical.

**Método:**

estudio transversal, realizado con pacientes prostatectomizados. Los datos fueron recogidos a partir de los siguientes instrumentos: cuestionario sociodemográfico, *Pad Test*, *International Consultation on Incontinence Questionnaire - Short Form y King Health Questionnaire*. Los datos fueron sometidos al análisis estadístico descriptivo y bivariado. El nivel de significancia adoptado fue de 0,05.

**Resultados:**

participaron 152 pacientes, con edad media de 67 años. Entre los pacientes incontinentes, hubo predominio de la incontinencia urinaria leve. La incontinencia urinaria causó impacto muy grave en la evaluación general de la calidad de vida en los primeros meses y grave después de seis meses de cirugía. Cuanto mayor la pérdida urinaria, mayor el impacto en los dominios de la calidad de vida Limitaciones Físicas, Limitaciones Sociales, Impacto de la Incontinencia Urinaria y Medidas de Gravedad. La mayoría de los participantes relató ausencia de erección después de la cirugía y por eso no respondió a la pregunta referente a la presencia de incontinencia urinaria durante la relación sexual.

**Conclusión:**

el estudio evidenció la ocurrencia de la incontinencia urinaria después de la prostatectomia radical en diferentes niveles y su impacto significativo en la calidad de vida de los hombres mereciendo intervenciones para su control.

## Introduction

Prostate cancer (PC) is the most incident in the male population, with the exception of non-melanoma skin cancer. The possibilities of treatment are surgery, radiotherapy and hormonal therapy. These options can cause undesirable effects, such as erectile dysfunction, loss of libido and urinary incontinence (UI), which also imply emotional changes, although temporarily^1^ ^-^ ^2^.

Radical prostatectomy (RP) is the gold standard treatment option for localized PC cases^3^. However, post-RP UI (PRPUI) may have a significant impact on men’s quality of life (QoL)^3^ ^-^ ^4^.

PRPUI is more severe in the early postoperative phase. However, continence recovery may occur within the first three to six months or belatedly (one year or more), as described in a meta-analysis that evaluated the effectiveness of exercises for pelvic muscles in the control of PRPUI^2^. Therefore, the male population may present different levels of urinary loss over time after surgery.

In turn, this problem may generate feelings of low self-esteem, anxiety, and depression that are usually present in men after surgery because of the uncertainty about how to deal with those undesirable effects^3^ ^-^ ^4^. A review that analyzed 19 studies concerning male UI identified that the different perceptions and reactions experienced determine the extent of emotional conflict, the difficulties faced in life and the self-perception of health^5^. The association of physiological, psychological and behavioral factors related to urinary control influences the QoL of prostatectomized men^3^ ^,^ ^5^.

Health-related QOL is a multidimensional construct that involves relevant aspects of the patient’s life, such as the health status in general, symptoms related to the treatment of the disease, physical capacity, psychological state and social factors^6^. In this context, prostatectomized men require attention and care regarding factors related to UI that impact their QoL.

As strategies for assessing the physiological ability to control urinary continence, there are the objective tests, such as urodynamic study, Pad Test and exercise test, which classify the degree of UI by the quantification of urinary loss^7^.

However, these parameters do not assess the impact of UI on the psychological and behavioral aspects from the patient’s perception. Therefore, questionnaires were designed to evaluate the subjective aspects of urinary dysfunction that may influence QoL^7^. The use of these instruments allows comparing the effects of different existing therapies and strategies to control urinary loss, an essential factor to optimize the actions not only of nurses but also of the whole multiprofessional team^8^.

Among the existing instruments for evaluation of the QoL associated with UI, we can mention the International Consultation on Incontinence Questionnaire - Short Form (ICIQ-SF) and the King Health Questionnaire (KHQ), both translated and validated for the Brazilian context^9^ ^-^ ^10^. The concomitant use of these questionnaires becomes relevant as the ICIQ-SF provides a brief and general assessment of the impact of UI on QoL while KHQ provides an assessment of the impact of UI on different QoL domains related to the physical, social and emotional aspects^9^ ^-^ ^10^.

Thus, knowing how the PRPUI can influence QoL enables the targeting of educational nursing actions and the referencing of these individuals for multiprofessional follow-up, and optimizes the communication between professional and patient with a focus on the problems experienced by these men.

With the intention of contributing to the knowledge in the area of PRPUI, the present study is relevant inasmuch it aims to evaluate the level of UI and its impact on the QoL of patients submitted to RP.

## Method

This is a cross-sectional study carried out with patients submitted to RP in a reference service in the state of Minas Gerais (MG), Brazil, linked to the National Cancer Institute (INCA), which has a team of urology surgeons who perform RP through the Unified Health System (SUS), health plan or private system.

The sample size was calculated from the population of men seen at the institution in a two-year interval, equivalent to 242 men, 5% of margin of error and a 95% confidence level, which resulted in a minimum size of 149 individuals^11^.

Participants were selected from the following inclusion criteria: adults having been submitted to RP in an interval of two months to two years, since studies point to a high rate of PRPUI reported from the second month after RP that may last up to two years^12^; preserved locomotor, visual, hearing and swallowing ability; and cognitive ability assessed through the Mini Mental State Examination^13^. Patients using an indwelling bladder catheter (IBC) and reporting UI prior to surgery were excluded.

Data collection was from December 2016 to August 2017. Data were collected by two previously trained researchers regarding interview techniques in order to guarantee standardization.

For purposes of sociodemographic characterization, an instrument was elaborated that contemplated data such as age, education, per capita income, profession, marital status and time after surgery.

In order to objectively quantify the urinary loss, we used the one-hour Pad, a resource validated and recommended by the International Continence Society (ICS)^14^. The test consists of placing a penile absorbent pad next to the external urethral meatus, which will be weighed on a high sensitivity scale after the interval of one hour. During this interval, patients are initially asked to drink 500 milliliters of water and wait at rest for 15 minutes. Then, they are subjected to a protocol that simulates activities of daily living. Finally, after one hour, the penile absorbent pad is removed, which allows the assessment of urinary loss by different sources - effort, urgency and overflow^14^. Urinary losses are classified as insignificant - losses up to 1 gram (g); mild loss - between 1.1 and 9.9 g; moderate loss -between 10 and 49.9 g; and severe loss - above 50 g. The Pad Test quantifies but does not distinguish the mechanism that led to this urinary loss^14^.

To evaluate the impact of UI in different domains of QoL, considering the physical, social and emotional aspects, we used the KHQ instrument, which presents Cronbach’s α coefficient of 0.7 in its original version^9^. In the present sample, the instrument presented Cronbach’s α coefficient of 0.9, which means high reliability. The instrument consists of 21 questions organized in ten domains and another nine related to the symptoms of UI, as well as a space for the patient to report any other problem associated with the functioning of the bladder. Thus, for all answers, numerical values are assigned, summed and evaluated by domain, obtaining a score that varies from zero to 100, in which the higher the value obtained, the worse the QoL^10^.

In order to complement the information obtained by KHQ, the ICIQ-SF instrument was also used for a general and brief evaluation of the impact of UI on QoL. The ICIQ-SF is a short, self-administered questionnaire composed of four questions that assess the frequency, severity and impact of UI, as well as eight items related to the causes and situations of UI experienced by the patient. The instrument was developed in the English language and in 2004 it was validated in the Portuguese language, presenting high psychometric capacity and Cronbach’s α coefficient of 0.9^9^. In the present sample, the instrument presented Cronbach’s α coefficient of 0.8. The total score is obtained by adding the scores of questions three, four and five, with values varying from zero to 21, considering that the higher the score obtained, the worse the QoL. Thus, the impact on QoL is classified according to the score: no impact (zero point); mild impact (one to three points); moderate impact (from four to six points); severe impact (from seven to nine points); and very severe impact (10 or more points)^9^.

Data were processed and analyzed using the Statistical Package for Social Science (SPSS), version 23.0 for Windows. The Shapiro-Wilk test was used to test the normality^11^ of the explanatory variables, being those with normal distribution presented by mean and standard deviation (SD), and the others in the median and interquartile range (p25-p75).

Three categories of post-surgery time were defined, namely group 01 (G1) - two to six months; group 02 (G2) - more than six months to one year; group 03 (G3) - more than one year to two years^2^. With the aim of evaluating the difference between the three groups of the weight of the absorbers used in the Pad Test, the one-way Anova test was applied. The analysis of the difference between the QoL domains of the KHQ questionnaire as a function related to the time after surgery also included the one-way Anova test followed by the post hoc analysis (Games-Howell and Gabriel’s), considering statistical difference for p-value less than 0.05. The Kruskal-Wallis test was also performed for non-normal distribution variables, followed by the 2 x 2 analysis to identify possible differences between the groups using the Bonferroni correction. The significance level corrected after this procedure was p <0.016^11^.

In order to identify possible relationships between the QoL domains measured by the KHQ and the urine loss assessed by the Pad Test, Pearson’s correlation test was applied. The strength of the correlations were analyzed considering values up to 0.30 as low magnitude, between 0.31 and 0.59 moderate, and above 0.60 of strong magnitude^11^ ^).^

The national ethical recommendations on human research provided by the National Health Council were met, and the project was approved by the ethics committee of the proposing institution, under opinion no. 1,866,160/2016.

## Results

The study sample consisted of 152 patients, 68 in G1, 40 in G2, and 44 in G3. In terms of age, the participants presented an average of 66.8 (± 7.8) years, ranging from 47 to 83 years. Regarding schooling, the participants presented an average of 3.9 (± 2.9) years of study, ranging from zero to 11 years. The average per capita income was 898.30 (± 525.70) reais, ranging from zero to 3,748.00 reais. Regarding the profession, 78.9% were inactive (retired or unemployed) and 21.1% were in active situation. As for the marital status, 80.3% of the participants had a partner. There was no statistically significant difference between groups G1, G2 and G3 for age (p = 0.394), schooling (p = 0,190), per capita income (p = 0.826), professional status (p = 0.547) and marital status (p = 0.951). 

Regarding the level of UI evaluated by the weight of the absorbent pad through the Pad Test, it was observed that the G1 presented a higher mean UI (5.7grams ± 13.7), followed by G3 (2.0 grams ± 3.7) and, finally, the G2 (2.0 grams ± 3.7). In turn, the difference was not statistically significant between the groups (p = 0.310).

Table 1 shows the UI classification by weight of absorbent pad.

**Table 1:** Classification of urinary incontinence by weight of absorbent pad during the one-hour Pad Test in men submitted to radical prostatectomy. Divinópolis, MG, Brazil, 2016 - 2017

UI* Classification	**General (%)**	**Group 01 (%)**	**Group 02 (%)**	**Group 03 (%)**
**Insignificant**	**53.3**	**42.6**	**62.5**	**61.4**
Mild UI*	**34.2**	**41.2**	**27.5**	**29.5**
Moderate UI*	**10.5**	**14.7**	**10.0**	**4.5**
Severe UI*	**2.0**	**1.5**	**0.0**	**4.5**

*UI - Urinary incontinence

In the classification of incontinent patients, the mild UI category is the most frequent among patients in the three groups, and severe UI is the most prevalent among G3 men (4.5).

In the analysis of the impact of UI on QoL by ICIQ-SF, G1 presented a mean of 9.6 (± 5.5), classified as very severe, G2 6.9 (± 5.5) and G3 7.3 ( ± 5.4), classified as severe. When comparing the mean between groups, there was a statistically significant difference in the impact of UI on QoL in the first six months after surgery (G1) compared to the period of six months to one year (G2) (p = 0.022).

Table 2 shows the QoL domains evaluated by the KHQ instrument related to the different post-surgery periods. 

**Table 2:** Quality of life domains evaluation by the King Health Questionnaire in different periods after radical prostatectomy surgery. Divinópolis, MG, Brazil , 2016 - 2017

**Quality of Life domains**	**Group 01**	**Group 02**	**Group 03**	**P-value**
	Mean±SD*/Median **(p** ^**†**^ **25 - p** ^**†**^ **75)**	Mean±SD*/Median **(p** ^**†**^ **25 - p** ^**†**^ **75)**	Mean±SD*/Median **(p** ^**†**^ **25 - p** ^**†**^ **75)**	
**Impact of urinary incontinence**	**46.1±38.2** ^**‡**^	**26.7±31.3** ^**‡**^	**31.1±32.5**	**0.011** ^**§**^
**Limitations of daily activities**	**19.8±31.0** ^**‡**^	**7.1±17.2** ^**‡**^	**9.8±21.1**	**0.023** ^**§**^
**Physical limitations**	**18.9±25.7**	**9.2±17.7**	**9.5±20.1**	**0.034** ^**§**^
**Severity Measures**	**32.3±26.7** ^**‡**^	**18.3±23.8** ^**‡**^	**18.7±26.3** ^**‡**^	**0.006** ^**§**^
**Sleep and mood**	**0.0 (0.0-16.7)**	**0.0 (0.0-0.0)**	**0.0 (0.0-29.2)**	**0.556** ^**||**^
**Perception of health**	**25.0 (25.0-50.0)**	**25.0 (25.0-50.0)**	**25.0 (0.0-25.0)**	**0.266** ^**||**^
**Social limitations**	**0.0 (0.0-22.2)** ^**‡**^	**0 (0-0)** ^**‡**^	**0 (0-0)** ^**‡**^	**0.027** ^**||**^
**Personal relationships**	**33.3 (0.0-50.0)**	**33.3(0.0-50.0)**	**41.7(16.7-50.0)**	**0.386** ^**||**^
**Emotions**	**0.0 (0.0-66.6)**	**0.0 (0.0-44.4)**	**0.0 (0.0-0.0)**	**0.222** ^**||**^

*SD - Standard deviation; ^†^p - percentile; ^‡^Statistically significant difference after multiple comparison of means according to the post-hoc test (p <0.05) or Bonferroni correction (p <0.016); ^§^One-way Anova test; ^||^Kruskal Wallis Test

When analyzing the QoL domains by the KHQ questionnaire, we observe statistically significant difference among the three groups in the domains Impact of UI, Limitations of Daily Activities, Physical Limitations, Severity Measures and Social Limitations. Considering Bonferroni’s correction to obtain greater clarity on the origin of the differences between the three groups, a statistically significant difference was identified between groups G1 and G2 in the domains Impact of UI (p = 0.014), Limitations of Daily Activities (p = 0.019) and Social Limitations (p = 0.012), the results being worse in G1 when compared to G2. The domain of Severity Measures, when controlled by type 1 error (Gabriel’s post-hoc test), showed a significant difference of G1 in relation to G2 (p = 0.020) and G3 (p = 0.021), that is, the QoL due to Severity Measures of UI (use of absorbent pads, limitation of fluid intake, need for changing wet underwear, shame, urine odor) is worse in the first six months after surgery. 

Table 3 shows the correlation forces between the QoL domains of the KHQ instrument and the urinary loss quantified by the Pad Test. 

**Table 3:** Pearson correlation between the quality of life domains of the King Health Questionnaire and urinary loss after radical prostatectomy. Divinópolis, MG, Brazil, 2016-2017

**Quality of Life domains**	**Correlation coefficient**	**P-value**
**Perception of health**	**-0.036**	**0.658**
Impacto of UI*	**0.305**	**<0.001**
**Limitation of Daily Activities**	**0.116**	**0.154**
**Physical Limitations**	**0.370**	**<0.001**
**Social Limitations**	**0.374**	**<0.001**
**Personal relationships**	**0.067**	**0.724**
**Emotions**	**0.096**	**0.237**
**Sleep and Moods**	**0.141**	**0.084**
**Severity Measures**	**0.447**	**<0.001**

*UI - Urinary incontinence

In the analysis of the correlation between the QoL domains of the KHQ and the urinary loss, the domains Impact of UI, Physical Limitations, Social Limitations and Severity Measures presented a positive relation of moderate magnitude with UI, that is, the greater the urinary loss, the greater their impact on these domains of QoL.

Table 4 presents the symptoms of UI assessed using the KHQ instrument according to the different postoperative periods. 

**Table 4:** Symptoms related to urinary incontinence in different periods after radical prostatectomy. Divinópolis, MG, Brazil, 2016 - 2017

**Symptoms**	**Group 01**			**Group 02**			**Group 03**		
	**A little %**	**Moderately** **%**	**Very much %**	**A little %**	**Moderately** **%**	**Very much %**	**A little %**	**Moderately** **%**	**Very much %**
**Urinary frequency**	**19.1**	**47.1**	**32.4**	**30.0**	**37.5**	**30.0**	**18.2**	**52.3**	**29.5**
**Nocturia**	**39.7**	**27.9**	**20.6**	**50.0**	**22.5**	**12.5**	**22.7**	**40.9**	**18.2**
**Urinary urgency**	**22.1**	**19.1**	**20.6**	**30.0**	**20.0**	**12.5**	**18.2**	**22.7**	**15.9**
**Unge-incontinence**	**32.4**	**16.2**	**11.8**	**32.5**	**10.0**	**7.5**	**36.4**	**13.6**	**4.5**
Stress UI*	**32.4**	**13.2**	**13.2**	**70.0**	**22.5**	**5.0**	**20.5**	**11.4**	**9.1**
**Nocturnal enuresis**	**4.4**	**13.2**	**4.4**	**10.0**	**10.0**	**2.5**	**11.4**	**4.5**	**2.3**
UI* during intercourse	**4.4**	**1.5**		**7.5**	**2.5**		**6.8**	**4.5**	
**Frequent urinary infections**	**17.6**			**27.5**	**2.5**	**2.5**	**18.2**	**9.1**	**2.3**
**Bladder pain**	**7.4**	**1.5**		**10.0**	**5.0**		**15.9**	**2.3**	

*UI - Urinary incontinence

It was observed that the symptoms that were very present in the three groups were urinary frequency (G1 = 32.4%, G2 = 30.0%, G3 = 29.5%), nocturia (G1 = 20, G2 = 12.5%, G3 = 18.2%) and urinary urgency (G1 = 20.6%, G2 = 12.5%, G3 = 15.9%). The symptoms classified as “very little present”, with less prominence in the three groups, were nocturnal enuresis (G1 = 4.4%, G2 = 10.0%, G3 = 11.4%), UI during sexual intercourse (G1 = 4, G2 = 7.5%, G3 = 6.8%) and bladder pain (G1 = 7.4%, G2 = 10.0%, G3 = 15.9%).

The item “UI during sexual intercourse” was not answered by 119 participants (78.3%), since they reported not having sexual intercourse due to the absence of erection.

## Discussion

The present study revealed that the one-hour Pad Test was able to detect involuntary loss of urine in participants of the three groups categorized according to the period after surgery. The most frequently reported incontinence loss classified as mild was in the period of two to six months after surgery, although there was no statistically significant difference between groups. Studies indicate a higher prevalence of UI in the first six months and a tendency to decreased urinary loss over time^2^ ^,^ ^12^ ^,^ ^15^, however, no studies were found describing the classification of the most frequent UI levels in this period.

Among patients with postoperative period of two to six months, UI had a very severe impact on QoL according to ICIQ-SF, whereas among patients with more than six months after surgery (G2 and G3) the impact on QoL was severe. Similar data were found in a Brazilian study that obtained an average of 10.6 points in the general QoL of incontinent post-RP patients in the third postoperative month and an average of 9.2 points in the sixth month, thus classifying the impact as very severe^15^. 

Regarding the QoL domains Impact of UI, Limitations of Daily Activities and Social Limitations, there was a greater impact in these domains in patients between two and six months postoperatively compared to those with more than six months to one year. In this sense, PRPUI significantly compromises the lifestyle of men. Anxiety caused by urinary loss interferes with QoL, restricts social and family contact, and generates feelings of loss of control of life. Patients report shame and discomfort due to the inability to control the bladder in the presence of their relatives and friends or for having to wear a diaper or having to change it out of home. There are also patients who do not wear a diaper daily, but always carry it for safety in their bag when they leave home, afraid of urinary leakage due to some physical effort^16^. UI, besides provoking feelings of loss of masculinity^16^, also threatens the decline in professional performance and household chores^5^.

The results of the study indicated statistical significance in the difference between groups for the impact of UI in the field of UI Severity Measures (use of absorbent pads, limitation of fluid intake, need for changing wet underwear, shame, urine odor), being more severe in the first six months. The literature corroborates this finding and points to a higher impact of UI in the QoL during the first months after surgery, mainly due to the need to use absorbent pads and the feeling of shame, with spontaneous and progressive improvement over time^2^ ^,^ ^5^ ^,^ ^16^.

In relation to the correlation results between the QoL and the UI level, there was a positive relationship of the urinary loss with the Impact of UI, Physical Limitations, Social Limitations and Severity Measure domains. Increased urinary loss implies an increase in the number of daily absorbent pads and this impacts on the patients’ perception of their QoL, since authors point out that patients who use one absorbent pad per day are considered continents and, therefore, have their QoL preserved when compared those who use two or more absorbent pads^17^.

Regarding the positive correlation of UI with the domains Physical and Social Limitations, it is possible to point out that acts resulting from the intensity of urinary loss, such as urine odor and wet clothes, affect the emotional and social aspect of these men because they feel stigmatized, generating an impaired self-image^5^. Such situation changes the lifestyle of these men, who start to take several baths a day, wear dark clothes, limit their physical activities for fear of urinary leakage, and even tend to isolate themselves^5^. 

It is important to consider that part of the impairment of mental well-being that affects QoL is related to the lack of social support, as well as to the fear associated with psychological and affective relationships, especially fear of being abandoned by wives, reported by at least half of patients^18^.

These results reinforce the need for health professionals to reflect and act on the problem in order to organize care that guides men with UI in a welcoming and individualized way, within the framework of the altered physical and psychosocial aspects.

The most prevalent UI symptoms at different post-PR periods in all groups were urinary frequency, nocturia, and urinary urgency. These symptoms are characteristic of detrusor overactivity and when associated with sphincter dysfunction represent the cause of 23% to 42% of cases of PRPUI^19^. Sphincter dysfunction may be developed by bladder devascularization or denervation, or by inflammatory changes related to surgery, or as a result of vesico-urethral reflex activation^19^.

Among the symptoms classified as “very little present”, the finding referring to the item “UI during sexual intercourse” was not answered by the majority of the participants (78.3%) due to the absence of an erection. Corroborating with the results found, researchers identified a rate of 83%^15^ and 85%^20^ of patients with erectile dysfunction. RP can reduce erectile function in up to 60% of patients undergoing surgery within two years^21^. During the surgical procedure, the vascular-nerve bundles and the smooth muscle can be affected, thus compromising the penile erection^4^. Researchers have identified that surgery negatively affects sexual function causing a significant impact in the social domain regarding the evaluation of post-RP QoL^15^. Another study also reinforces this finding by pointing out that the impact on erectile dysfunction goes beyond the physical aspect and affects psychological and relationship aspects^20^.

The literature offers important suggestions for increasing the QoL of patients with PRPUI, such as cognitive-behavioral therapy in training of coping skills, support and informative groups, interventions for altered body image, expression and regulation of emotions, as well as conservative therapies that can optimize the time the patient needs to return to be continent^18^. Thus, it is possible to minimize the negative impact of cancer and UI on the QoL of individuals.

A review study aimed at exploring men’s perception on the impact of the physical consequences of RP on their QoL suggests that improving QoL should be the ultimate goal of any treatment or intervention, in which the successful experience of the patient with the treatment is a prime factor. In addition, the results of the review also recommend the development of psychoeducational interventions related to UI and erectile dysfunction before and after RP to increase men’s understanding and adaptation to these postoperative symptoms^22^.

In this context, nursing acts directly in the care of patients submitted to RP, both physically and psychologically, and plays a fundamental role in providing adequate preparation for RP and the potential implications in postoperative QoL^18^. Knowing and understanding men’s experiences after RP provides healthcare professionals with the ability to provide comprehensive support and information that is vital to these patients^22^.

When analyzing the results of the present study, one should consider the sample profile, characterized by low level of education, and the possibility of not perceiving urinary loss and its real impact due to lack of knowledge about the physiology of their own body. Ignorance may also be associated with the embarrassment and shame of men in expressing their perception of urinary loss. Culturally, this group may also interpret urinary loss as a natural part of the aging process, since it is predominant in the elderly. Therefore, such factors may limit a more reliable fulfillment of the subjective instruments ICIQ-SF and KHQ. Thus, it is important to reproduce studies in samples with different sociodemographic profiles, also in relation to the level of schooling, for amplification and greater generalization of the results. 

## Conclusion

The results showed that the urinary loss evaluated by the Pad Test had a predominance of mild UI among incontinent patients in the three groups. UI had a very severe impact on overall QoL assessment in the first six months and severe impact after six months of surgery. The domains of QoL that presented a statistically significant difference between the three groups were Impact of UI, Limitations of Daily Activities, Physical Limitations, Severity Measures and Social Limitations. It was also identified that the greater the urinary loss, the greater the impact in the areas Physical Limitations, Social Limitations, Impact of UI and Severity Measures. Most participants reported no erection after surgery and therefore did not answer to the question on the presence of urinary incontinence during sexual intercourse.

We hope that these findings instigate the need to implement multifaceted interventions based on a better understanding of the physical and emotional implications of the patient undergoing RP. Nurses have a major role in subsidizing the planning and implementation of actions that aim to improve the urinary continence of these individuals and, consequently, their QoL.
